# Assessment of bone graft incorporation by ^18^ F-fluoride positron-emission tomography/computed tomography in patients with persisting symptoms after posterior lumbar interbody fusion

**DOI:** 10.1186/2191-219X-2-42

**Published:** 2012-07-30

**Authors:** Boudewijn Brans, Rene Weijers, Serve Halders, Roel Wierts, Marloes Peters, Ilona Punt, Paul Willems

**Affiliations:** 1 Department of Nuclear Medicine, University Medical Center Maastricht, Postbox 5800, Maastricht, 6202 AZ, The Netherlands; 2 Department of Radiology, University Medical Center Maastricht, Postbox 5800, Maastricht, 6202 AZ, The Netherlands; 3 Department of Orthopedic Surgery, University Medical Center Maastricht, Postbox 5800, Maastricht, 6202 AZ, The Netherlands; 4 University Hospital Geneva, Rue Gabrielle-Perret-Genti 4, Geneva 14, 1211, Switzerland; 5 Research School CAPHRI, University Maastricht, Postbox 616, Maastricht, 6200, MD, The Netherlands

**Keywords:** F-fluoride, PET/CT, spinal fusion, lumbar spine, bone stress, subsidence

## Abstract

**Background:**

Posterior lumbar interbody fusion (PLIF) is a method that allows decompression of the spinal canal and nerve roots by laminectomy combined with fusion by means of intervertebral cages filled with bone graft and pedicle screw fixation. Conventional imaging techniques, such as plain radiography and computed tomography (CT), have limitations to assess bony fusion dynamics.

**Methods:**

In 16 PLIFs of 15 patients with persisting symptoms, positron-emission tomography (PET)/CT scans were made 60 min after intravenous administration of 156 to 263 MBq of ^18^ F-fluoride, including 1-mm sliced, high-dose, non-contrast-enhanced CT scanning. Maximal standard uptake values (SUVmax) of various regions were calculated and correlated with abnormalities on CT.

**Results:**

Subsidence of the cages into the vertebral endplates was the most frequently observed abnormality on CT (in 16 of 27 or 59% of evaluable endplates). Endplate SUVmax values were significantly higher for those patients with pronounced (*p* < 0.0001) or moderate (*p* < 0.013) subsidence as compared to those with no subsidence. Additionally, a significant correlation between vertebral and ipsilateral pedicle screw entrance SUVmax values (*p* < 0.009) was found, possibly indicating posterior transmission of increased bone stress. In our patient group, intercorporal fusion was seen on CT in 63% but showed no correlation to intercorporal SUVmax values.

**Conclusions:**

With the use of ^18^ F-fluoride PET/CT, intervertebral cage subsidence appeared to be a prominent finding in this patient group with persisting symptoms, and highly correlating with the degree of PET hyperactivity at the vertebral endplates and pedicle screw entry points. Further study using ^18^ F-fluoride PET/CT should specifically assess the role of metabolically active subsidence in a prospective patient group, to address its role in nonunion and as a cause of persisting pain.

## Background

Low back pain is a major health care problem affecting up to 80% of the population in industrial countries [1]. In the Netherlands, it was estimated that the direct health care costs exceed €700 million, and the indirect costs related to lost productivity, due to absence from work and early retirement, are ten times higher [2]. A main cause of low back pain is lumbar spondylolisthesis, with a prevalence of up to 6% in the general population. In spondylolisthesis, there is an anterior slip of usually the fourth or fifth lumbar vertebra caused by either a lysis in the pars intervertebralis of the lamina or by disc degeneration. In the case of intractable low back pain and/or radiating leg pain, caused by narrowing of the neural foramina, surgical decompression and fixation may be indicated. The goal of operative treatment is to decompress entrapped nerve roots and to stabilize the slipped vertebra. A frequently performed treatment of the segmental instability caused by low-grade lumbar spondylolisthesis (0% to 50% anterior slip of the upper vertebra) is posterior lumbar interbody fusion (PLIF). PLIF involves the insertion of intervertebral cages filled with autologous bone into the disc space after removal of the intervertebral disc, combined with partial or complete resection of the lamina and facet joints, and pedicle screw fixation of the two vertebrae providing primary stability. Definite bony interbody fusion is generally achieved in 91.1% of cases after a minimal follow-up of 24 months, corresponding to a clinical success in 83% [3]. A failure of fusion of the vertebral bodies by inadequate bone formation, a so-called ‘pseudarthrosis,’ can lead to persisting lumbar instability and pain.

Standard postoperative assessment of lumbar interbody fusion includes lateral flexion-extension radiographs to detect intervertebral motion. In patients with persistent or recurrent symptoms, thin (1 to 2 mm)-sliced computed tomography (CT) with coronal and sagittal reconstructions to examine the presence of bridging bone has been advocated [4]. A CT scan can very well exclude a pseudarthrosis (negative predictive value of 100%) [5], but the positive predictive value for pseudarthrosis is only 21%, as compared to the gold standard of surgical re-exploration. Moreover, current imaging techniques, such as plain radiography and CT, cannot reliably assess bony fusion at an early stage. Only in case of complete trabecular bony bridging, ingrowth as a sign of complete segmental fusion can be confirmed which, in general, takes about a year to occur. In addition, the presence of intervertebral cages makes it harder to assess fusion reliably. So, in case bony ingrowth in cages has to be assessed, plain radiography and CT are not of clinical use in the early postoperative phase.

It has been stated that radiological lesions with corresponding increased bone scan activity are lesions that alter skeletal metabolic activity and may therefore be of clinical importance. Functional changes as evident on radionuclide techniques may precede structural anatomical changes on CT [6]. This suggests the potential usefulness of nuclear medicine techniques in these patients. With the detector sensitivity and spatial resolution superior to single photon emission computed tomography (SPECT)/CT, ^18^ F-fluoride positron-emission tomography (PET)/CT can provide a quantitative evaluation of blood flow, bone stresses, and remodeling of bone in intervertebral disc spaces and facet joints [7,8]. In the only published study so far on this topic, Fischer and co-workers [9] found a persistent activity around intervertebral cages in 48% of cages after more than 1 year between surgery and PET/CT, suggesting unsuccessful fusion due to increased stresses, overcharge, and microinstability.

The aim of the present study was to quantify vertebral ^18^ F-fluoride uptake patterns in correlation with findings on high-resolution CT images with regard to the process of interbody fusion, in patients with persisting symptoms after PLIF.

## Methods

### Patients

A cohort of 15 patients was enrolled in this study between June 2008 and January 2011. Inclusion to the study group was done on the basis of (1) the surgical technique, i.e., a PLIF with pedicle screw fixation, and (2) the clinical presentation, i.e., patients in whom PLIF had been performed and low back pain persisted or recurred without an obvious clinical explanation. Time interval between fusion surgery and the PET/CT examination was 4 to 31 months (mean 16 months, median interval 13 months). This study is part of a research protocol that has been accepted by the medical ethical committee of the University Medical Center Maastricht (NL.32881.068.11) and in which patients give their informed and written consent.

### Posterior lumbar interbody fusion

Through a posterior lumbar approach, the nerve roots were decompressed by laminectomy or laminotomy and the intervertebral disc was excised. After thorough cleansing of the endplates, one or two 10- to 12-mm-thick carbon fiber cages (PEEK®, Medtronic, Memphis, TN, USA), filled with autologous bone from the lamina, were inserted into the disc space. In addition, the upper and lower vertebrae were fixed by transpedicular screws for primary stabilization. In one patient, PLIFs were inserted at two vertebral levels, so a total of 16 PLIFs were evaluated.

### ^18^ F-fluoride PET/CT scans

Sixty minutes after intravenous injection of 156 to 263 MBq (mean 199 MBq, median 196 MBq) of ^18^ F-fluoride, PET and CT images were made with an integrated PET/CT scanner (Gemini TF PET-CT, Philips, Amsterdam, The Netherlands). After a low-dose CT acquisition (120 kV, 30 mAs, slice thickness 4 mm), a PET scan was made in three-dimensional mode, by acquiring two bed positions of 5 min, covering the lumbosacral spine. This was immediately followed by a high-dose, non-contrast-enhanced CT scan (64-slice helical, 120 kV, 250 mAs, slice 1 mm with increment of 0.8 mm) of the fusion region. Standard filtered backprojection CT reconstruction was performed. PET images were reconstructed both non-attenuated and CT-based attenuated using time-of-flight technology. Images were postprocessed and viewed on custom software (EBW, Philips, Eindhoven, The Netherlands).

### PET/CT evaluation

CT-attenuation-corrected and non-attenuation-corrected PET and high-dose CT images were analyzed independently by two experienced observers blinded from the results of previous imaging and unaware of clinical details. Maximal standard uptake values (SUVmax) were calculated in various subregions, using volumes of interest drawn on the corresponding transaxial, sagittal, and coronal CT slices, encompassing the whole of the following subregions: upper and lower vertebral endplates, right and left sides of these, entrance point and tip of the pedicle screws, and facet joints. Verification of these values was done by visual inspection of the CT-attenuation-corrected as well as non-attenuation-corrected PET images.

**Figure 1:**
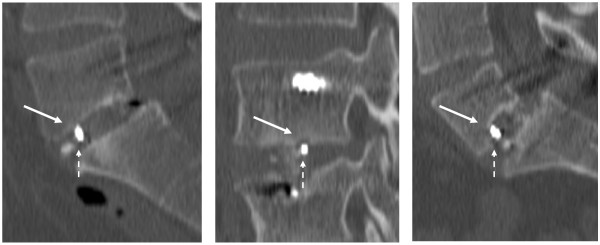
**Vertebral subsidence.** Examples of no (left), moderate (middle), and pronounced (right) vertebral subsidence, as assessed on CT by the degree of sinking of the intervertebral cage into the vertebral body (closed arrow) and the position of the radiological markers of the intervertebral cage (open arrow).

Signs of implant failure or loosening, migration, or subsidence of the cages were evaluated on plain radiographs and CT scan. Intervertebral fusion was defined by the presence of an intervertebral bone bridge on both sides (*positive or score 2*), on one side, right or left (*intermediate or score 1*), or no bridging (*negative or score 0*). Subsidence of the intervertebral cages, as assessed on plain radiographs and CT, was scored as no (*score 0*), moderate (*score 1*), or pronounced subsidence (*score 2*) into the right and/or the left side of either the upper or lower vertebrae or both (Figure 1). Subsidence of the upper and lower vertebrae were separately scored, hence a total of 32 values.

### Statistical evaluation

Statistical evaluation was performed using SPSS software version 18.0 (IBM Corporation, Armonk, NY, USA). A test for normality of distribution was done according to a one-sample Kolmogorov-Smirnov testing. Comparison of continuous SUVmax values with postoperative interval was done with a parametric paired-sample *t* test. Possible associations between SUVmax values of vertebrae, pedicles, and facet joints were tested using a multivariate analysis of variance (ANOVA). Comparison of the SUVmax statistics between subgroups of fusion and subsidence grade was made using a one-way ANOVA, with post-hoc multiple comparisons done according to Tukey. Correlations between anterior (vertebral) and posterior (pedicle) values were assessed according to Pearson. *P* values < 0.05 were considered statistically significant.

## Results

### Patient characteristics

Table 1 shows the main characteristics of patients.

**Table 1:** Patient characteristics

**Gender/age**	**Indication**	**Level**	**Cages**	**Interval (months)**
F24	Lysis-olisthesis	L5-S1	2	22
F51	Discopathy	L3-L4	1	24
F65	Degenerative olisthesis	L4-L5	2	10
F65	Discopathy	L3-L4	2	12
M55	Lysis-olisthesis	L5-S1	2	12
F49	Lysis-olisthesis	L4-L5	2	7
M39	Discopathy	L4-L5; L5-S1	1	24
M48	Lysis-olisthesis	L5-S1	2	6
F41	Lysis-olisthesis	L5-S1	2	10
M46	Lysis-olisthesis	L5-S1	2	4
F41	Lysis-olisthesis	L5-S1	2	17
F33	Lysis-olisthesis	L5-S1	2	14
M50	Lysis-olisthesis	L5-S1	2	26
F19	Lysis-olisthesis	L4-L5	2	31
M49	Lysis-olisthesis	L5-S1	2	6

The mean age of six men and nine women was 46 years old, with a range of 19 to 65 years. The indication for PLIF was predominantly a low-grade (i.e., less than 50% slip of the upper vertebra) spondylolisthesis on the basis of spondylolysis, i.e., a congenital breach in the processus articulares, in 11 of 15 cases. Minor indications were a listhesis or discopathy or on the basis of degenerative disease (4 of 15 cases). The level of surgery was mainly L5-S1. Time interval between fusion surgery and the PET/CT examination was 4 to 31 months (mean 16 months, median 13 months).

### PET findings

Visually increased activity on PET scan was observed in 7 of 15 (46%) patients in the upper endplates and in 7 of 15 (46%) patients in the lower involved endplates. Additionally, we saw focally increased activity specifically at the entry points of the pedicle screws in 11 patients (73%). In no case did we see focally increased activity further along the track of the pedicle screw. In 6 patients (40%), one or more facet joints were hyperactive. The most pronounced hyperactivity on the PET scan was observed at the entry point of one of the pedicles in 7 of 15 (46%) patients, at an endplate of the vertebrae in 5 of 15 (33%), at a facet joint in 3 of 15 (20%), and in the intervertebral fusion area in 1 of 15 (7%).

**Figure 2:**
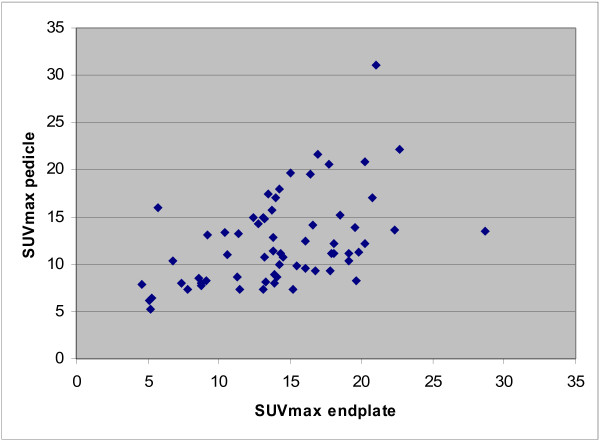
^**18**^**F-fluoride bone activity distribution after PLIF.** Correlation between upper, lower, right, and left endplate PET activity with corresponding pedicle screw entry point PET activity.

**Figure 3:**
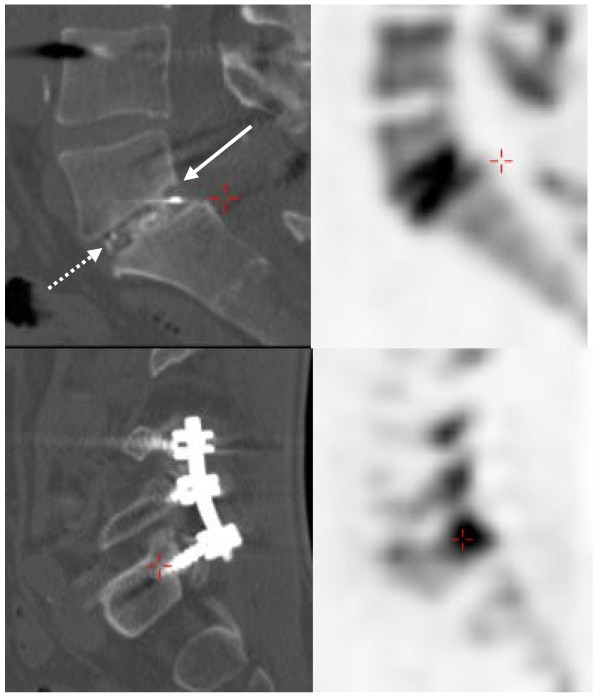
**PET/CT after PLIF spondylodesis.** Example of a patient, 7 months after PLIF for a lysis-olisthesis of L5-S1. Moderate subsidence on CT (upper left, closed arrow) and vertebral endplate activity on PET (upper right). Associated pedicle screw entrance point hyperactivity on PET (lower right, cross), possibly indicating continued bone stress post-surgery. The intervertebral area (dotted arrow) shows the bone fragments inside the cage and one of its posterior radiological markers.

Figure 2 shows the comparison between SUVmax values at the right and left sides of the upper and lower vertebral endplates with their corresponding values at the entry point of four pedicle screws. Multivariate ANOVA demonstrated a significant association not only between the upper and lower endplate values (*p* < 0.007), but also between the values on the right and left sides of the endplate and their corresponding ipsilateral pedicle values (*p* < 0.009). The correlation between these values was also statistically significant (Pearson correlation coefficient of 0.473, *p* < 0.01). Figure 3 shows an example of this. These findings did not alter if the SUVmax values were normalized by the SUVmax values of adjacent, uninvolved vertebrae, or if values from non-attenuation-corrected images were used.

### PET/CT findings

**Figure 4:**
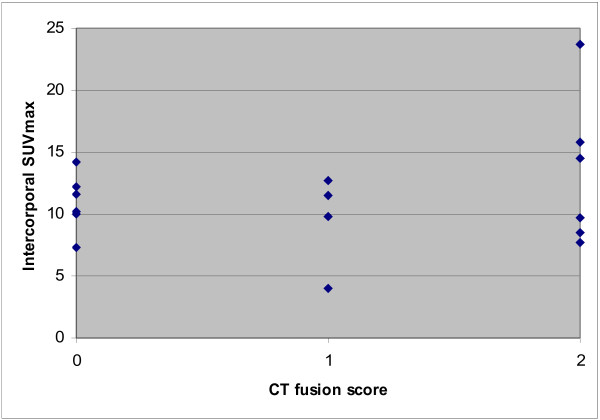
^**18**^**F-fluoride activity in vertebral fusion.** Intervertebral SUVmax values with corresponding fusion scores on CT.

Intercorporal fusion was found in 6 of 16 (38%) PLIFs on both right and left sides, in 4 of 16 (25%) on one side only, and in 6 of 16 (38%) on neither side. Figure 4 shows the relation with the SUVmax values in these groups. No statistical significant differences between these groups and their intercorporal SUVmax values were found, nor with regard to postoperative intervals or with the age of the patient.

**Figure 5:**
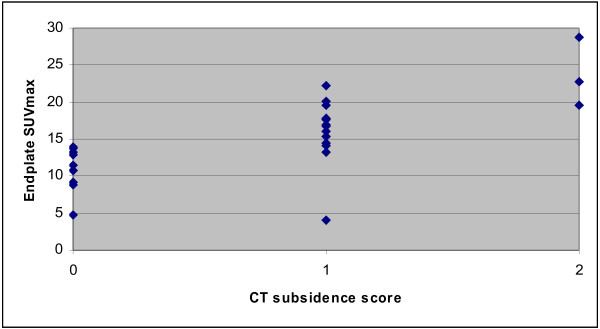
^**18**^**F-fluoride activity in vertebral subsidence.** SUVmax values of vertebral upper and lower endplates within corresponding subsidence scores on CT.

Vertebral endplate subsidence was the most frequent CT abnormality. In 5 of 32 endplates, no certain distinction between grades 1 and 2 could be made with the available quality of images; these values were discarded. Pronounced subsidence was seen in 3 of 27 (11%); moderate subsidence, in 13 of 27 (48%); and no subsidence, in 10 of 27 (37%). Figure 5 shows the SUVmax values in relation to the categories of subsidence, as evident on CT. A significant difference was found between the values of the no subsidence group compared to those of the moderate (*p* < 0.013) and pronounced (*p* < 0.0001) subsidence groups, as well as between the subsidence groups (*p* < 0.015). This was unrelated to the length of the postoperative interval, i.e., there was no statistical association between the SUVmax values of the upper and lower endplates with the postoperative interval in months. Pedicle screw abnormalities were less often seen on CT: 2 of 16 (13%) PLIFs with loosening of a screw and 2 PLIFs with screw breakage. Additionally, three cases of screw malposition through the facet joint were found. In two of these three cases, this was associated with a hotspot on PET. In two other cases, CT indicated degenerative changes in the facet joint with no corresponding PET hyperactivity. Other incidental findings included cases of vertebral hemangioma, kissing spine, transitional S1 vertebra, and active sacroiliitis.

## Discussion

The most salient finding of this study was an increased uptake of ^18^ F-fluoride at the endplates of the upper and lower vertebral bodies of the fusion region, which was significantly associated with the degree of cage subsidence on CT, regardless of the length of follow-up. Subsidence can be defined as a decrease in height of the disc space at follow-up as compared to immediately postoperative [10], or more specifically, as a sinking of the cages into the vertebral body, resulting in a counterproductive decrease in disc height and neuroforamina caliber with increasing risk of recurrent neurological symptoms, pedicle screw failure, and pseudarthrosis. Whether subsidence occurs or not is subject to many factors, such as composition, shape, and size of the cage; thickness and extent of the resected endplates; and vertebral bone mineral density. It is important that the intervertebral cages are capable of dispersing the compressive interbody forces uniformly over the endplates [11]. Partial removal of the endplates at surgery, intended to improve vascularization into the intercorporal fusion area, weakens the compression resistance of the vertebral bodies [12]. Whether subsidence is an inherent phenomenon of nonunion or should be regarded as an evitable complication is presently under debate. Some researchers have not found a correlation between radiographic fusion and recurrence of symptoms with the development of subsidence [13]. Subsidence produces a local and general kyphotisation and destabilization which increases bone stress at the screw-bone interface [14]. This may explain the association that we found between activity at the endplates and at the entry points of the screws in the pedicle. In our series, we observed two cases of screw breakage and two cases of loosening, which may have been the complication of this and seems more than reported in the literature [15]. However, one has to acknowledge that our patient group was biased towards patients with persisting/recurrent pain, and thus a higher rate of implant failure may be expected.

There are sparse reports in the nuclear medicine literature regarding the postoperative evaluation of spinal surgery. Using ^99m^Tc-imidodiphosphonate SPECT, Even-Sapir et al. [16] studied a mixed group of fusion techniques 2 to 21 years after surgery. They identified abnormalities in the free-moving vertebral bodies and apophyseal joints above and below the fusion area as a source of increased bone stress and pain. Gates and McDonald [17] found a high degree of facet abnormalities in a mixed group of predominantly simple laminectomy without fusion, suggesting that decompression without fixation creates an instability of the posterior arcus and hence stress and arthropathy of the facet joints. Recently, Damgaard et al. [18] used SPECT-CT in nine postoperative patients with pain and detected loose pedicle screws in six patients. Using ^18^ F-fluoride PET/CT, Fischer et al. [9] recently observed an increased activity around intervertebral cages as compared to surrounding bone in 14 of 29 (48%) cages >1 year after surgery. A significant difference was found in the median time interval between patients with and without increased uptake, with a median time of 22 months and 100 months, respectively. They interpreted this increased uptake as indicative of ‘unsuccessful fusion due to increased stress and microinstability.’ Interestingly, the PET/CT image provided as an example in their article shows a similar ‘traintrack’-like appearance of endplate hyperactivity that we predominately found in our patients, suggesting that active subsidence was also present in at least some of their patients.

The findings from the present pilot study are limited by the small, clinically heterogeneous patient sample with a variable time interval after surgery. Additionally, surgical re-exploration as a gold reference standard was not performed. In our institution, second-look exploration is only performed in cases of clearly diagnosed complications, renewed instability, and/or intractable pain. Only one patient underwent revision surgery due to screw breakage, and perioperatively, pseudarthrosis was confirmed. Nevertheless, this study highlights the phenomenon of subsidence as a cause of persisting hperactivity following post-operative PET/CT. For clinical decision making, it is important to know whether metabolically active subsidence is indicative of nonunion and may be regarded as the cause of persisting pain after PLIF surgery. The inherent specificity of ^18^ F-fluoride uptake implies that other causes of increased uptake such as the surgical procedure itself or underlying bone pathology such as spondylolisthesis or secondary spondyloarthrosis should be distinguished. Based on the results of the current study, a prospective clinical study using sequential scanning and inclusion of both symptomatic and asymptomatic PLIF patients has been initiated.

## Conclusions

With the use of ^18^ F-fluoride PET/CT, intervertebral cage subsidence appeared to be correlated with the degree of PET hyperactivity at the vertebral endplates and pedicle screw entry points. This suggests nonunion with instability as the source of pain in patients with persisting symptoms after lumbar interbody fusion. PET/CT may offer valuable insights in device design by demonstrating patterns of bone stress during incorporation. The excellent resolution and quantification of PET/CT may help to address the clinical relevance of vertebral subsidence in patients with persisting pain after spinal interbody fusion.

## Abbreviations

CT, computed tomography; 18F, 18fluor; PET, positron-emission tomography; PLIF, posterior lumbar interbody fusion; SPECT, single photon emission computed tomography; SUVmax, maximum standardized uptake value.

## Competing interests

The authors declare that they have no competing interests.

## Authors’ contributions

BB designed the study and performed the data analysis. RW performed the data analysis. SH performed the data acquisition and data processing. RW performed the data processing and data analysis. MP performed the literature search and data analysis. PW designed the study, performed the patient recruitment, data acquisition, and data analysis, and coordinated the study. All authors participated in the manuscript drafting and approval. All authors read and approved the final manuscript.

## Authors’ information

The authors take part in a multidisciplinary academic working group developing new imaging modalities and applications for orthopedic prosthesis imaging. BB is a senior nuclear medicine physician with years of experience in skeletal scintigraphy, SPECT, and PET. RW is an acknowledged musculo-skeletal specialist in radiology. RW is a junior physicists with an interest in PET and SPECT processing. SH is a highly experienced nuclear medicine radiographer. MP is a junior researcher with a background in technical and computer skills. IP is a post-doctorate researcher in osteoprosthesis and motion analysis. PW is a senior orthopedic surgeon specialized in advanced spine surgery.
